# Choline attenuates NEFA-induced hepatic steatosis via GNMT regulation in hepatocytes

**DOI:** 10.1007/s44154-025-00264-3

**Published:** 2025-11-14

**Authors:** Xueer Du, Lamei Wang, Yanfei Dai, Jing Lu, Hongrui Li, Dangdang Wang, Jun Zhang, Chuanjiang Cai, Shimin Liu, Junhu Yao, Jianguo Wang, Yangchun Cao

**Affiliations:** 1 College of Animal Science and Technology, Northwest A&F University, Yangling, 712100 China; 2 UWA Institute of Agriculture, The University of Western Australia, Crawley, WA Australia; 3 College of Veterinary Medicine, Northwest A&F University, Xianyang, 712100 China

**Keywords:** Perinatal dairy cows, Lipid deposition, NEFA, Choline, GNMT

## Abstract

To elucidate the molecular mechanisms by which choline regulates hepatic lipid metabolism under negative energy balance conditions, we established non-esterified fatty acid (NEFA)-induced hepatic steatosis models in both calf primary hepatocytes and human LO2 hepatocytes. Choline supplementation significantly reduced intracellular triglyceride accumulation and cytotoxicity induced by NEFA exposure. Transcriptomic profiling identified glycine N-methyltransferase (GNMT) as a key differentially expressed gene. Subsequent experiments confirmed that choline upregulated GNMT expression at both the mRNA and protein levels in a concentration-dependent manner. Knockdown of GNMT reversed the beneficial effects of choline on genes related to lipid synthesis (FAS, ACC), fatty acid oxidation (CPT1), lipoprotein assembly (ApoB100, MTTP), and bile acid metabolism (CYP7A1, CYP27A1, BSEP). Furthermore, inhibition of AMP-activated protein kinase (AMPK) reduced GNMT protein expression and elevated Myc, a negative transcriptional regulator of GNMT, suggesting that choline may regulate GNMT through the AMPK/Myc axis. Collectively, our findings demonstrate that choline alleviates NEFA-induced lipid accumulation and hepatocellular damage by modulating lipid and bile acid metabolism through GNMT, with the AMPK/Myc/GNMT signaling axis playing a pivotal regulatory role. These results provide mechanistic insights into the hepatic protective effects of choline and suggest GNMT as a potential therapeutic target for metabolic disorders in dairy cows and beyond.

**Supplementary Information:**

The online version contains supplementary material available at 10.1007/s44154-025-00264-3.

## Introduction

Periparturient dairy cows often experience a state of NEB, where energy demands for lactation exceed dietary intake (Zhou et al. 2016a; Shi et al. 2020). This metabolic imbalance drives the mobilization of adipose tissue, releasing large quantities of NEFA into circulation (Duttaroy and Basak 2021). The liver, as the primary organ for energy homeostasis, uptakes these NEFAs and either oxidizes them for energy production or re-esterifies them into TG, contributing to lipid storage (Zom et al. 2011; Goselink et al. 2013). However, the hepatic oxidative capacity is frequently insufficient to process excessive NEFAs during the perinatal period, leading to TG accumulation, impaired very-low-density lipoprotein (VLDL) secretion, and ultimately hepatic steatosis—a condition commonly observed in periparturient cows and associated with reduced productivity and increased disease risk (Sun et al. 2016; Huang et al. 2019).

To mitigate hepatic lipid overload, nutritional interventions have been explored, among which choline supplementation has shown promising effects (Wiedeman et al. 2018b). Choline is a quaternary amine essential for phospholipid synthesis, lipoprotein assembly, methyl-group donation, and one-carbon metabolism (Korsmo et al. 2019). In ruminants, its efficacy is enhanced through rumen-protected choline (RPC) formulations, which have been demonstrated to improve liver function, increase milk production, and attenuate NEB-associated lipid disorders (Lima et al. 2012). Mechanistically, choline has been reported to modulate lipid metabolism by enhancing VLDL export (via ApoB100, MTTP) and promoting fatty acid oxidation (via CPT1), while also participating in the synthesis of S-adenosylmethionine (SAM) through oxidation to betaine (Zom et al. 2011; Elek et al. 2013; Goselink et al. 2013; Ringseis et al. 2015). Furthermore, choline functions as a methyl donor in the one-carbon cycle, potentially mitigating disease progression and improving milk yield and quality in dairy cows (Zhou et al. 2016b; Fling et al. 2020; Holdorf et al. 2023). Despite these functional benefits, the precise molecular mechanisms through which choline exerts its hepatoprotective effects remain incompletely defined. In particular, the role of glycine N-methyltransferase (GNMT)—a key regulator of the SAM/S-adenosylhomocysteine (SAH) ratio and methyl donor flux—in mediating choline’s effects on lipid and bile acid metabolism has not been fully elucidated. Additionally, upstream signaling pathways such as AMP-activated protein kinase (AMPK) and its interaction with the transcriptional repressor Myc may influence GNMT expression and function under metabolic stress.

In this study, we established NEFA-induced hepatic lipid accumulation models in calf primary hepatocytes and human LO2 cells to investigate the regulatory effects of choline on hepatocellular lipid metabolism. Through transcriptomic analysis, gene knockdown, and pathway interrogation, we identified GNMT as a critical downstream effector of choline and examined the potential involvement of the AMPK/Myc/GNMT axis. Our findings provide mechanistic insights into choline-mediated attenuation of hepatic steatosis and identify potential molecular targets for improving metabolic health in dairy cows during the periparturient period.

## Results

### Choline reduces NEFA-induced hepatic lipid accumulation and cytotoxicity in calf primary hepatocytes

**Fig. 1:**
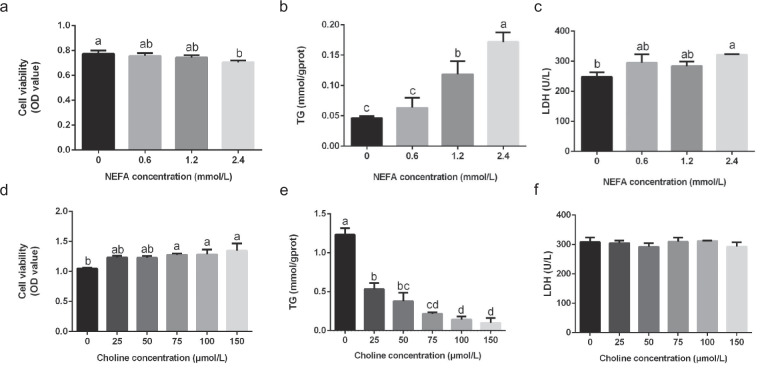
NEFA-induced lipid deposition model and choline-mediated regulation in primary calf hepatocytes. **a**-**c** Effects of different concentrations of NEFA (0, 0.6, 1.2, 2.4 mM) on (**a**) cell viability (CCK-8 assay), (**b**) TG accumulation, and (**c**) LDH activity in calf primary hepatocytes. **d**-**f** Modulation by choline (0, 25, 50, 75, 100, 150 μM) under 1.2 mM NEFA exposure on (**d**) cell viability, (**e**) TG content, and (**f**) LDH activity in the established lipid deposition model. Each treatment was performed in triplicate with independent biological replicates. Data are presented as mean ± SEM. Lowercase letters indicate statistically significant differences (*P* < 0.05, Duncan’s test)

To establish a hepatic steatosis model, calf primary hepatocytes were treated with increasing NEFA concentrations (0, 0.6, 1.2, and 2.4 mM). At 2.4 mM, NEFA significantly reduced cell viability cell viability (*P* < 0.05 vs. control), accompanied by a marked increase in LDH release (*P* < 0.05) and intracellular TG content (*P* < 0.05). In contrast, 1.2 mM NEFA markedly elevated TG content (*P* < 0.05 vs. control) without significantly compromising viability or LDH activity (*P* > 0.05), suggesting it as an optimal condition to model pathological lipid accumulation while preserving cellular integrity (Fig. 1a-c).

In order to explore the therapeutic potential of choline in NEFA-exposed hepatocytes, primary calf Liver cells treated with 1.2 mM NEFA were supplemented with choline at concentrations ranging from 0 to 150 μM. It Cell viability significantly improved in the 75, 100, and 150 μM choline groups compared to the NEFA-only control (*P* < 0.05). TG content decreased significantly in all choline-treated groups (*P* < 0.05 vs. NEFA control), with hierarchical reductions observed across doses: the 25 μM group exhibited higher TG levels than the 75–150 μM groups (*P* < 0.05), and the 50 μM group retained more TG than the 100–150 μM cohorts (*P* < 0.05). LDH levels remained unchanged across groups (*P* > 0.05) (Fig. 1e-f). Based on these findings, 75 μM choline dose was selected for further studies due to its optimal balance between significant metabolic improvement (viability rescue, TG reduction) and relevance to cell physiological conditions.

### Transcriptomic analysis identifies GNMT as a potential regulatory factor for choline response

**Fig. 2:**
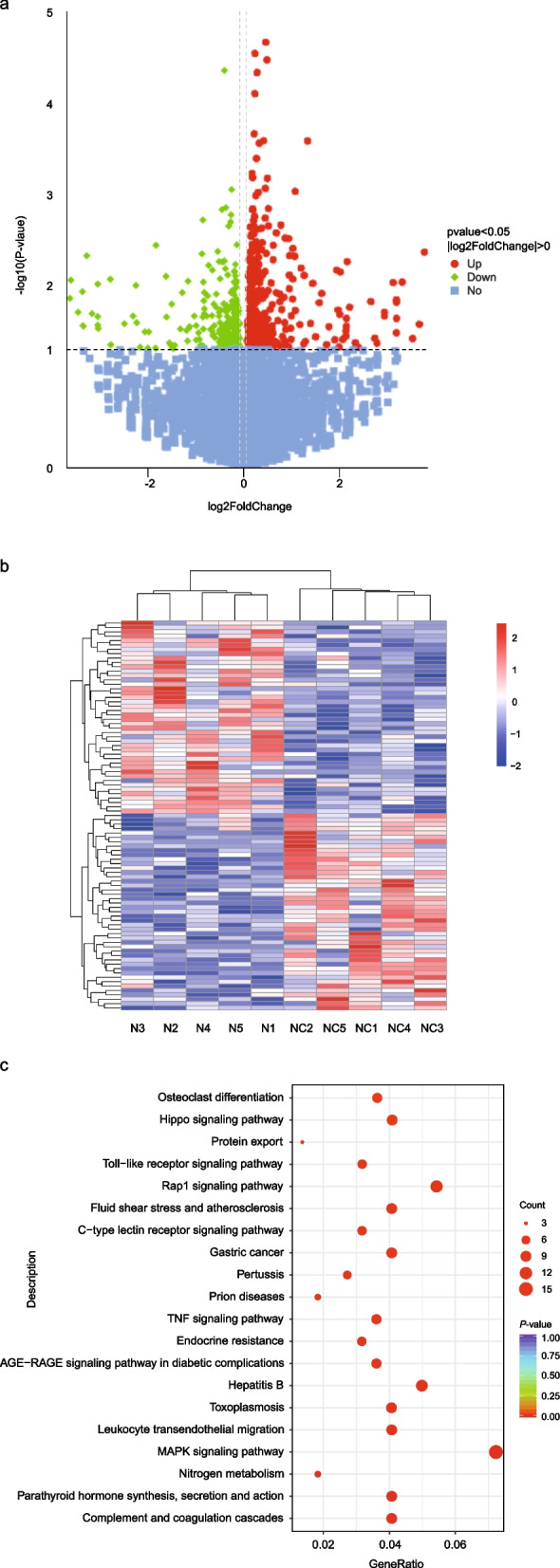
Transcriptomic profiling of calf primary hepatocytes under choline treatment. **a** Volcano plot of DEGs. **b** Clustered heatmap of DEGs (Top150). **c** KEGG enrichment pathways of DEGs

To elucidate the regulatory effects of choline on fat deposition under NEFA treatment, transcriptomic sequencing was performed on in calf primary hepatocytes samples from both a control group (0 μM choline) and a treatment group (75 μM choline). Following rigorous quality control (Table S3), the sequencing data were confirmed to be reliable for further analysis. Differential expression analysis identified a total of 480 DEGs between the two groups, with 249 genes upregulated and 231 downregulated (Fig. 2a, Table S4). Moreover, cluster heatmap analysis revealed distinct gene expression clusters between the groups (Fig. 2b).

Table 1 presents the top 30 DEGs exhibiting the most pronounced fold changes. In parallel, a comprehensive GO enrichment analysis was conducted on all DEGs between the NC and N groups, resulting in the annotation of 550 significantly enriched GO terms (*P* < 0.05) spanning the three principal categories: BP, CC, and MF (Table 2). GO enrichment analysis highlighted pathways related to oxidative stress response, MAPK cascade, vitamin biosynthesis, phosphate ion transport, etc. KEGG pathway analysis of DEGs revealed significant enrichment in 38 distinct pathways, and indicated enrichment in lipid metabolism, immune signaling (e.g., TNF, Toll-like receptor), and mechanotransduction pathways (Fig. 2c).

**Table 1:** Top 30 differentially expressed genes

Gene name	Description	Log_2_FC (NC/N)	*P*-value
HBA	hemoglobin, alpha 2	-3.47868	0.01981
VMO1	vitelline membrane outer layer 1 homolog	-3.37728	0.02763
U1	U1 spliceosomal RNA	-3.36998	0.02806
RANBP3L	RAN binding protein 3 like	3.31203	0.00927
MEIOC	meiosis specific with coiled-coil domain	3.19531	0.02317
SLC18A1	solute carrier family 18 member A1	3.18513	0.03328
C15H11orf16	chromosome 15 C11orf16 homolog	-3.06250	0.00976
CIITA	class II major histocompatibility complex trans activator	-3.05061	0.02968
CFAP52	cilia and flagella associated protein 52	2.93994	0.01987
SNORA70	small nucleolar RNA SNORA70	2.93738	0.02182
KIF26B	kinesin family member 26B	-2.80123	0.00868
RBM20	RNA binding motif protein 20	-2.79325	0.04513
TMEM196	transmembrane protein 196	2.73476	0.03820
KCNJ16	potassium inwardly rectifying channel subfamily J member 16	2.65060	0.01515
CCDC102B	coiled-coil domain containing 102B	2.39071	0.04799
LCTL	lactase like	-2.26608	0.01012
NKX2-2	NK2 homeobox 2	-2.25481	0.02570
FAM216B	family with sequence similarity 216 member B	-2.17846	0.04799
PPDPFL	pancreatic progenitor cell differentiation and proliferation factor like	2.15877	0.00553
VAT1L	vesicle amine transport 1 like	2.14020	0.02311
KCND2	potassium voltage-gated channel subfamily D member 2	2.13720	0.02821
HEMGN	hemogen	-2.07875	0.03236
PLIN4	perilipin 4	2.06558	0.03925
bta-mir-2346	bta-mir-2346	-2.01992	0.04866
GDF10	growth differentiation factor 10	2.01439	0.00720
BGLAP	bone gamma-carboxyglutamate protein	-2.01374	0.04912
NTRK1	neurotrophic receptor tyrosine kinase 1	1.99005	0.04762
ATP6V1G2	ATPase H + transporting V1 subunit G2	1.96545	0.01881
DNAAF1	dynein axonemal assembly factor 1	1.93793	0.00684
KCNT1	potassium sodium-activated channel subfamily T member 1	-1.84319	0.00367

*Abbreviations*: *N* 1.2 mM NEFA group, *NC* 1.2 mM NEFA + 75 μM choline group

**Table 2:** Top 40 GO enrichment items of DEGs

GO ID	Category	Description	*P*-vlaue	Counts
GO:0001503	BP	ossification	7.05E-08	24
GO:0001649	BP	osteoblast differentiation	3.72E-07	16
GO:0005791	CC	rough endoplasmic reticulum	2.92E-05	7
GO:0030278	BP	regulation of ossification	4.23E-05	14
GO:0044070	BP	regulation of anion transport	5.56E-05	8
GO:0009791	BP	post-embryonic development	7.05E-05	9
GO:0009110	BP	vitamin biosynthetic process	9.20E-05	5
GO:0010035	BP	response to inorganic substance	0.00011	18
GO:0032964	BP	collagen biosynthetic process	0.00012	5
GO:0045667	BP	regulation of osteoblast differentiation	0.00014	10
GO:0042359	BP	vitamin D metabolic process	0.00020	4
GO:0071496	BP	cellular response to external stimulus	0.00022	15
GO:0048592	BP	eye morphogenesis	0.00022	11
GO:1,901,652	BP	response to peptide	0.00038	16
GO:0048593	BP	camera-type eye morphogenesis	0.00044	9
GO:0033280	BP	response to vitamin D	0.00048	4
GO:0000302	BP	response to reactive oxygen species	0.00051	10
GO:0032963	BP	collagen metabolic process	0.00053	7
GO:0042542	BP	response to hydrogen peroxide	0.00059	7
GO:0001666	BP	response to hypoxia	0.00071	11
GO:0043410	BP	positive regulation of MAPK cascade	0.00073	20
GO:0036293	BP	response to decreased oxygen levels	0.00075	11
GO:0005844	CC	polysome	0.00077	6
GO:0035455	BP	response to interferon-alpha	0.00078	4
GO:0009612	BP	response to mechanical stimulus	0.00093	9
GO:0050905	BP	neuromuscular process	0.00095	8
GO:0042788	CC	polysomal ribosome	0.00096	4
GO:0001890	BP	placenta development	0.00114	9
GO:0034599	BP	cellular response to oxidative stress	0.00114	12
GO:0006817	BP	phosphate ion transport	0.00119	4
GO:0048596	BP	embryonic camera-type eye morphogenesis	0.00119	4
GO:0070301	BP	cellular response to hydrogen peroxide	0.00124	6
GO:0030282	BP	bone mineralization	0.00139	8
GO:0050858	BP	negative regulation of antigen receptor-mediated signaling pathway	0.00145	4
GO:0070167	BP	regulation of biomineral tissue development	0.00147	7
GO:0042368	BP	vitamin D biosynthetic process	0.00148	3
GO:0040017	BP	positive regulation of locomotion	0.00149	21
GO:0070482	BP	response to oxygen levels	0.00152	11
GO:0002237	BP	response to molecule of bacterial origin	0.00160	12
GO:0006979	BP	response to oxidative stress	0.00163	15

*Abbreviations*: *N* 1.2 mM NEFA group, *NC* 1.2 mM NEFA + 75 μM choline group

Among DEGs, several lipid-related genes were modulated by choline, notably *GDF10*, *IGFBP1*, *CREBRF*, *HSD17B8*, *TGFB3*, *GNMT, HSD17B8*, *TGFB3*, *ABCD1* (Table 3). Given its central role in one-carbon metabolism and methyl donor regulation, *GNMT* was selected for functional validation.

**Table 3:** DEGs related to fat and energy metabolism

Gene name	Description	Log_2_FC (NC/N)	*P*-vlaue	Up/Down
GDF10	growth differentiation factor 10	2.01439	0.00720	↑
PIWIL2	Piwi Like RNA-mediated silencing 2	-1.52559	0.04626	↓
IGFBP1	insulin Like growth factor binding protein 1	0.61307	0.00366	↑
CREBRF	CREB3 regulatory factor	0.53829	0.03631	↑
FGF23	fibroblast growth factor 23	0.48452	0.03898	↑
GNMT	glycine N-methyltransferase	0.45862	0.02978	↑
HSD17B8	hydroxysteroid 17-beta dehydrogenase 8	0.25903	0.00041	↑
TGFB3	transforming growth factor beta 3	0.21750	0.02151	↑
ABCD1	ATP binding cassette subfamily D member 1	0.20947	0.01760	↑

Log_2_FC (NC/N) > 0 was considered as upregulation of gene expression (↑), while Log₂FC (NC/N) < 0 was considered as downregulation (↓)

*Abbreviations*: *N* 1.2 mM NEFA group, *NC* 1.2 mM NEFA + 75 μM choline group

### GNMT exhibits biphasic regulation by choline in hepatic steatosis models

**Fig. 3:**
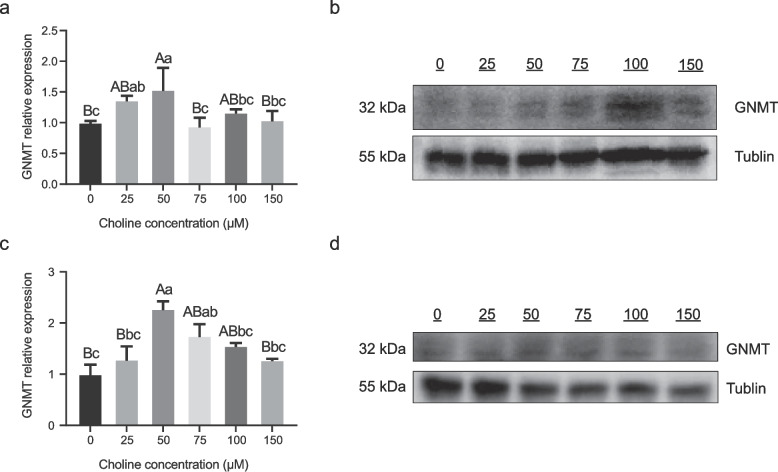
Impact of varying choline concentrations on GNMT expression in NEFA-induced hepatic lipid deposition models. **a**-**b** Effect of different choline concentrations on GNMT mRNA and protein expression in calf primary hepatocytes treated with 1.2 mM NEFA. **c**-**d** Effect of different choline concentrations on GNMT mRNA and protein expression in LO2 hepatocytes treated with 1.6 mM NEFA. Data are presented as mean ± SEM. Each treatment was performed in triplicate with independent biological replicates. Different lowercase letters denote significant differences at *P* < 0.05, whereas different uppercase letters indicate significance at *P* < 0.01 (Duncan’s test)

To assess the responsiveness of *GNMT* to choline, primary calf hepatocytes and LO2 cells were treated with NEFA and varying choline concentrations (0, 25, 50, 75, 100, and 150 μM). In primary calf hepatocytes, 50 μM choline significantly increased in GNMT mRNA levels compared to the 100 μM group (*P* < 0.05), and showed even greater upregulation relative to the 0, 75, and 150 μM groups (*P* < 0.01). Additionally, cells treated with 25 μM choline exhibited significantly higher GNMT expression than those treated with 0 and 75 μM (*P* < 0.05). No significant differences were observed among the remaining groups (*P* > 0.05) (Fig. 3a-b). These findings indicate a biphasic response in *GNMT* expression, with upregulation at moderate choline concentrations followed by a decline at higher doses.

A parallel experiment was conducted in the LO2 human hepatocyte lipid deposition model. First, a gradient of NEFA concentrations was used to induce steatosis, revealing a dose-dependent decrease in cell viability and corresponding increases in LDH activity and TG content in the culture medium (Fig. S1). Based on these results, 1.6 mM NEFA was selected as the optimal concentration to establish the Lipid deposition model. Cells were then exposed to the same gradient of choline concentrations. As observed in calf hepatocytes, 50 μM choline significantly enhanced *GNMT* mRNA expression compared to 100 μM (*P* < 0.05), and exhibited markedly higher levels relative to 0, 25, and 150 μM groups (*P* < 0.01). Furthermore, 75 μM choline also significantly upregulated GNMT expression compared to untreated controls (*P* < 0.05), with no significant differences among the other groups (*P* > 0.05) (Fig. 3c-d). In both calf primary hepatocytes and LO2 cells, *GNMT* expression showed a biphasic response. Western blot analysis confirmed protein-level consistency with transcript patterns, suggesting dose-sensitive regulation of GNMT by choline.

### GNMT knockdown alters lipid and bile acid metabolism in LO2 cells

To examine the functional role of GNMT in choline-mediated metabolic regulation, LO2 hepatocytes were transfected with GNMT-specific siRNA and exposed to NEFA (1.6 mM) with or without 50 μM choline. Knockdown efficiency was confirmed by reduced *GNMT* mRNA and protein expression (> 40%, *P* < 0.01) (Fig. S2a-b).

To assess the impact of GNMT knockdown on fat and bile acid metabolism, the mRNA expression levels of genes involved in fat synthesis, metabolism, cholesterol transport, and bile acid synthesis were evaluated in LO2 hepatocytes (Fig. S2c-d). The results revealed that, Compared to the NEFA-only group (N), GNMT knockdown (N-si) significantly increased expression of lipogenesis, oxidative genes and bile acid transpot genes (*FAS*, *ACC*, *CPT1*, *HMGCR*, *NTCP*, *BSEP*; *P* < 0.05) while downregulating lipid transport and bile acid synthesis genes (*ApoB100*, *PPARα*, *CYP7A1*, *CYP27A1*; *P* < 0.05).

**Fig. 4:**
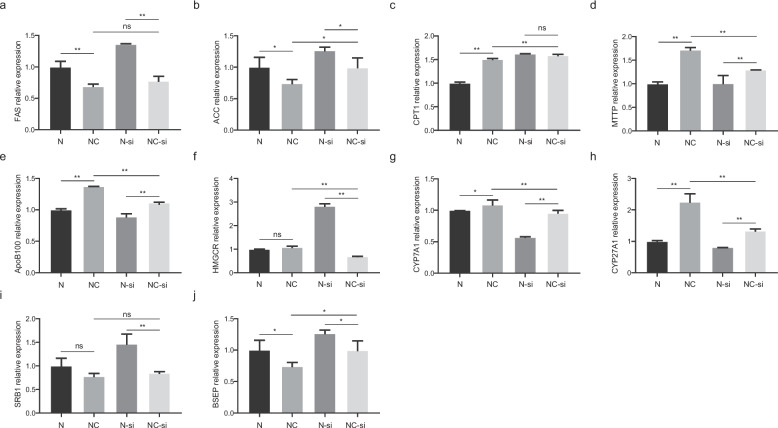
Impact of choline on lipid and bile acid metabolism in LO2 hepatocytes following GNMT knockdown under NEFA exposure. **a**-**e** Expression of lipid metabolism-related genes (FAS, ACC, CPT1, MTTP, ApoB100, and PPARα) in response to choline (50 μM) supplementation under 1.6 mM NEFA treatment with or without GNMT knockdown. **f**-**j** Expression of bile acid metabolism-related genes (HMGCR, CYP7A1, CYP27A1, SRB1, and BSEP) under 1.6 mM NEFA treatment with or without GNMT knockdown. Each treatment was performed in triplicate with independent biological replicates. Data are presented as mean ± SEM. Abbreviations: *N* = 1.6 mM NEFA; NC = 1.6 mM NEFA + 50 μM choline; N-si = 1.6 mM NEFA + GNMT-siRNA; NC-si = 1.6 mM NEFA + 50 μM choline + GNMT-siRNA. Statistical significance: *P < 0.05, **P < 0.01, ns = not significant (*P* > 0.05) (Duncan’s test)

To further examine whether choline’s effects on hepatic metabolism are mediated through GNMT, additional comparisons were made between the NC and NC-si groups. Compared to the N group, Choline supplementation (NC) reversed NEFA-induced dysregulation, downregulating *FAS* (*P* < 0.01), *ACC* (*P* < 0.05) and upregulating *CPT1*, *MTTP*, *ApoB100*, *CYP7A1*, *CYP27A1*, and *BSEP* (*P* < 0.05). Additionally, SRB1 expression exhibited a decreasing trend (0.05 < *P* < 0.1) (Fig. 4a-e). When GNMT was silenced in the NC-si group, we observed that compared with the N-si group, *FAS* and *ACC* expression were significantly reduced (*P* < 0.05), whereas *HMGCR* and *SRB1* levels were also markedly decreased (*P* < 0.01). Meanwhile, the expression of *MTTP*, *ApoB100*, *CYP7A1*, and *CYP27A1* was significantly increased (*P* < 0.05). However, relative to the NC group, GNMT knockdown in the NC-si group led to a significant increase in *ACC* expression (*P* < 0.05) and a marked decrease in *MTTP* and *ApoB100* expression (*P* < 0.05) (Fig. 4f-j). No statistically significant differences were observed in the remaining genes (*P* > 0.05). These results indicate that choline plays a pivotal role in regulating lipid and bile acid metabolism in hepatocytes.

### Choline regulates GNMT expression through the AMPK/Myc axis in adipose-deposited hepatocytes under NEFA exposure

To explore whether AMPK and MYC, a known negative regulator of GNMT, mediate the effect of choline on GNMT expression in adipose-deposited Liver cells under NEFA exposure, we constructed an adipose-deposited model using a calf primary hepatocytes Line exposed to 1.2 mM NEFA. AMPK inhibition was achieved by adding Compound C (10 μM), and the phosphorylation levels of AMPK as well as the gene expression of MYC were evaluated.

**Fig. 5:**
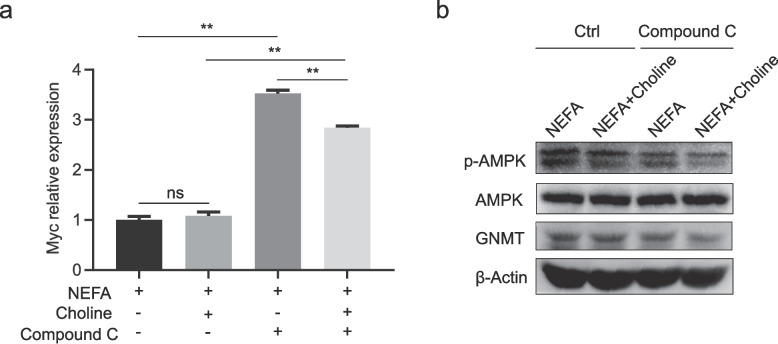
Verification of choline regulation of hepatic lipid metabolism via GNMT under AMPK inhibition. **a** Effect of choline (75 μM) on MYC gene expression in calf primary hepatocytes expression under AMPK inhibition (Compound C, 10 μM). **b** Effect of choline (75 μM) on protein expression levels of AMPK and GNMT in the presence of AMPK inhibitor (Compound C, 10 μM). Each treatment was performed in triplicate with independent biological replicates. Data are presented as mean ± SEM. Statistical significance: *P < 0.05, **P < 0.01, ns = not significant (*P* > 0.05) (Duncan’s test)

Inhibition significantly reduced AMPK phosphorylation and GNMT protein levels, while increasing Myc gene expression (*P* < 0.05) (Fig. 5a-b). Choline supplementation partially rescued GNMT expression despite AMPK inhibition and attenuated Myc upregulation, though not fully to control levels (*P* < 0.01). These results suggest that choline upregulates GNMT via AMPK activation and suppression of Myc, supporting the involvement of the AMPK/Myc/GNMT signaling axis in hepatic lipid metabolism under NEFA stress.

## Discussion

During the periparturient period, dairy cows commonly experience negative energy balance (NEB), which results in excessive lipolysis and elevated circulating non-esterified fatty acids (NEFA). The liver plays a central role in handling this lipid influx, but excessive NEFA accumulation surpasses its metabolic capacity, leading to hepatic steatosis and functional impairment (Contreras and Sordillo 2011). Under physiological conditions, NEFA are oxidized or exported as VLDL, but during NEB, impaired apolipoprotein synthesis results in hepatic lipid accumulation and functional impairment (McArt et al. 2013; Ringseis et al. 2015). Choline is essential for maintaining membrane integrity and function through its roles in phospholipid biosynthesis and apolipoprotein regulation, thereby promoting lipid transport and preventing hepatic lipid accumulation (Li and Vance 2008; Wiedeman et al. 2018a; Bernhard et al. 2019). While previous studies have focused on choline’s effects on lipid export and oxidation, the molecular mediators through which choline exerts these functions have not been clearly defined.

Our in vitro data demonstrate that NEFA exposure reduces hepatocyte viability and promotes triglyceride accumulation, whereas choline supplementation mitigates these effects by enhancing cell survival and reducing Lipid deposition. Transcriptomic analysis identified 480 choline-responsive genes, with functional enrichment highlighting metabolic and oxidative stress-related pathways such as MAPK, Ras, and FoxO signaling. These findings suggest that choline participates in broader regulatory networks beyond methyl donation.

A key observation in this study is the biphasic response of GNMT to choline, with moderate concentrations enhancing GNMT expression and higher doses dampening its induction. GNMT, a central regulator of one-carbon metabolism, SAM/SAH homeostasis, and mitochondrial function, was shown to mediate choline’s effects on hepatic lipid metabolism (Weinhold and Sanders 1973; Zom et al. 2011; Martínez-Uña et al. 2013; Cahova et al. 2015; Wang et al. 2015; Fernández-Ramos et al. 2018; Simile et al. 2018; Fernández-Tussy et al. 2019; Murray et al. 2019). Knockdown of GNMT disrupted the expression of genes related to lipogenesis (FAS, ACC), β-oxidation (CPT1), and lipid export (MTTP, ApoB100), supporting its central regulatory role.

Moreover, key enzymes such as ACC and FAS drive de novo lipogenesis, promoting triglyceride accumulation (Menendez and Lupu 2007; Koundouros and Poulogiannis 2020), while CPT1 regulates fatty acid β-oxidation by mediating mitochondrial fatty acid transport (Virmani et al. 2015). MTTP and ApoB100 facilitate VLDL assembly and lipid export, and PPARα enhances fatty acid oxidation during lipid mobilization (Smati et al. 2020; Peng et al. 2021). GNMT knockdown in steatotic hepatocytes upregulated FAS, ACC, and CPT1, but downregulated ApoB100 and PPARα, highlighting GNMT’s role in balancing lipid synthesis and export. Although choline suppressed lipogenesis (FAS, ACC) and promoted oxidation and export (CPT1, MTTP, PPARα), these effects were blunted in GNMT-deficient cells—particularly for CPT1—emphasizing GNMT as a key mediator of choline's regulatory influence on hepatic lipid metabolism.

Cholesterol homeostasis is closely intertwined with hepatic lipid metabolism. HMGCR governs cholesterol synthesis, while SR-B1 mediates HDL uptake (Shen et al. 2014). Cholesterol is primarily catabolized into bile acids via CYP7A1 (classical pathway) and CYP27A1 (alternative pathway), with transporters such as BSEP and NTCP maintaining bile acid balance and hepatic function (Russell 2009; Li and Apte 2015; Ticho et al. 2019; Chiang and Ferrell 2020). GNMT knockdown in NEFA-treated hepatocytes upregulated HMGCR and downregulated CYP7A1, CYP27A1, and NTCP, indicating enhanced cholesterol synthesis and impaired bile acid metabolism. Although choline modulated these genes, the partial recovery of CYP7A1, CYP27A1, and BSEP suggests its effects may also involve alternative regulatory pathways such as FXR or TGR5 signaling (Holter et al. 2020; Huang et al. 2022). Future studies should investigate GNMT’s subcellular localization and post-translational modifications on transcriptional regulation using techniques such as ChIP-seq and methylome analysis.

AMPK is a central regulator of mitochondrial and metabolic homeostasis, inhibiting lipid and cholesterol synthesis by phosphorylating ACC and HMGCR, and modulating transcription factors (SREBP1, ChREBP, and HNF4) (Kawaguchi et al. 2002; Hong et al. 2003; Li et al. 2011). AMPK activation reduces malonyl-CoA via ACC inhibition, thereby relieving its suppression of CPT1 and enhancing fatty acid oxidation (Fullerton et al. 2013). Inhibition of AMPK phosphorylation significantly upregulated Myc—a transcription factor involved in proliferation and apoptosis—and concurrently reduced GNMT protein levels in NEFA-treated cells, even with choline supplementation (Zheng et al. 2017; Kfoury et al. 2018; Kant et al. 2019; Ahmadi et al. 2021). These findings suggest that choline exerts its regulatory effect on GNMT through the AMPK/Myc axis. Despite these findings, in vitro models cannot fully recapitulate the complexity of the periparturient bovine liver, including hormonal fluctuations, inflammatory signals, and systemic lipid mobilization. Thus, in vivo studies are necessary for physiological validation.

## Conclusion

**Fig. 6:**
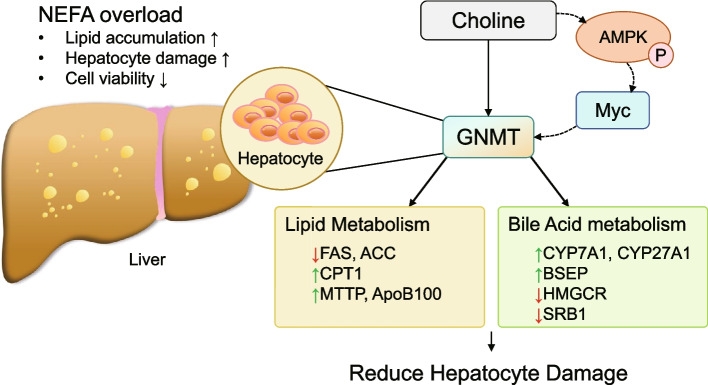
Mechanism of Choline-Mediated Alleviation of NEFA-Induced Hepatic Steatosis via the AMPK/Myc/GNMT Axis. Choline alleviates NEFA-induced hepatic steatosis via GNMT upregulation, mediated in part by AMPK-dependent suppression of Myc. GNMT regulates lipridation, lipoprotein assembly,and bile acid synthesis

This study reveals that choline supplementation mitigates NEFA-induced hepatic lipid accumulation by activating glycine N-methyltransferase (GNMT), which regulates key genes involved in fatty acid oxidation, lipoprotein transport, and bile acid metabolism. Mechanistically, GNMT is modulated through the AMPK/Myc axis, positioning it as a crucial effector of choline’s hepatoprotective action.These results provide novel insights into choline’s metabolic function and identify GNMT as a promising regulatory node for managing lipid disorders in dairy cows and potentially in broader contexts of hepatic steatosis. Further in vivo studies will help to validate these findings and clarify the multifunctional roles of GNMT in liver physiology (Fig. 6).

## Methods and materials

### Reagents and cell culture media

A NEFA stock solution (10 mM total) was prepared by dissolving palmitic acid (3.19 mM), palmitoleic acid (0.53 mM), stearic acid (1.44 mM), linoleic acid (0.49 mM), and oleic acid (4.35 mM) (Sigma-Aldrich, USA) in 113 mL of 0.1 M KOH at 60℃. After complete dissolution, 1.0 M HCl (pre-warmed to 60℃) was added to adjust the pH to 7.0–7.4, followed by dilution with 67.8 mL of ultrapure water. With complete mixing, the solution was cooled to room temperature, aseptically filtered through a 0.45 μm sterile filter, aliquoted, and stored at -20℃ (Liu et al. 2020; Deng et al. 2021).

Three types of RPMI-1640 media (HyClone, USA) were used:

Complete medium: RPMI 1640 medium was supplemented with 10% FBS (Gibco, BRL) and 1% penicillin/streptomycin solution (Sigma-Aldrich, USA).

Choline-free starvation medium: RPMI 1640 medium was used without choline and supplemented with 3.6% BSA (Amersco) and 1% penicillin/streptomycin solution (Sigma-Aldrich, USA).

Choline-free complete medium: RPMI 1640 medium was prepared without choline, containing 10% FBS (Gibco BRL), 3.6% BSA (Amersco), and 1% penicillin/streptomycin solution (Sigma-Aldrich, USA).

Choline Chloride: Choline chloride (Sigma-Aldrich, USA) was used as the source of choline in this study, which is commonly used in both the feed industry and cell culture research.

Further details on the preparation of the choline-free RPMI 1640 medium can be found in Supplement Material 1.

### Cell isolation and culture

Calf primary hepatocytes were isolated from calf liver using a three-step perfusion method (Supplementary Material 2) (Zhang et al. 2012). The isolated calf primary hepatocytes and human LO2 cells were resuspended in complete medium and seeded into a 6-well plate at a density of 1.5–2 × 10⁶ cells/mL and cultured at 37℃ in 5% CO₂; medium was refreshed every 24 h. Further details can be found in Supplement Material 2.

### Hepatocyte treatment

Prior to treatment, cells were incubated in choline-free starvation medium for 6 h. Thereafter, cells were switched to choline-free complete medium and treated with NEFA at various concentrations to establish Lipid deposition models. For calf hepatocytes, NEFA concentrations of 0, 0.6, 1.2, and 2.4 mM were tested, while LO2 cells were exposed to 0, 0.4, 0.8, 1.2, 1.6, and 2.4 mM. Optimal conditions were determined based on effects on cell viability, intracellular TG accumulation, and LDH release. Choline was supplemented at gradients (0, 25, 50, 75, 100, and 150 μM) to assess its effect on Lipid deposition. Samples were collected 12 h post-treatment for primary hepatocytes and 24 h for LO2 cells.

GNMT Knockdown: LO2 cells were transfected with GNMT-specific siRNA using a standard lipofection protocol, yielding four groups: N: 1.6 mM NEFA; NC: 1.6 mM NEFA + 50 µM choline; N-si: 1.6 mM NEFA + GNMT-siRNA; NC-si: 1.6 mM NEFA + 50 µM choline + GNMT-siRNA. Transfection was performed in choline-free complete medium; medium was replaced with fresh complete medium after 4–6 h. Cells were starved for 6 h and cultured for an additional 24 h before sample collection. All reagents were purchased from Gene Pharma, Shanghai, China.

AMPK Inhibition Assay: To investigate the role of AMPK in the regulation of lipid metabolism, Compound C (Selleck, Houston, USA) was added to the calf primary hepatocytes line. Four experimental groups were established: N (1.6 mM NEFA); NC (1.6 mM NEFA + 75 µM choline); N + AMPK inhibitor (1.6 mM NEFA + 10 µM Compound C); NC + AMPK inhibitor (1.6 mM NEFA + 75 µM choline + 10 µM Compound C). Cells were harvested after 12 h for gene and protein analyses.

### Quantitative real-time PCR

Total RNA was extracted from cells using TRIzol reagent (Invitrogen, Thermo Fisher, USA) following the manufacturer's instructions. Approximately 1–2 µg of RNA was treated with DNase (Invitrogen, Thermo Fisher, USA) to remove genomic DNA and subsequently reverse transcribed into complementary DNA (cDNA) using M-MLV Reverse Transcriptase (Accubate Biology, Changsha, China). Quantitative real-time PCR (RT-PCR) was performed using SYBR® Select Master Mix (Accubate Biology, Changsha, China) and the Roche LightCycler® 480 Real-Time PCR System. Relative gene expression was calculated using the 2^−∆∆Ct^ method, with normalization to the housekeeping genes β-actin and GAPDH (Supplement Tables 1 and 2).

### Western blotting

Proteins were extracted using lysis buffer (Proteintech, Chicago, USA). The prepared proteins were separated by SDS-PAGE using 10% (w/v) polyacrylamide gels and transferred onto PVDF membranes (Proteintech, Chicago, USA) using a wet transfer system. The membranes were blocked with 4% bovine serum albumin (BSA) in Tris–HCl buffer for 2 h at room temperature, followed by overnight incubation at 4℃ with primary antibodies against GNMT, AMPK, p-AMPK, and β-actin (ABclonal, Wuhan, China). After primary antibody incubation, the membranes were washed three times for 5–8 min each with TBS containing 0.1% Tween 20. Subsequently, the membranes were incubated with appropriate secondary antibodies (ABclonal, Wuhan, China) for 1.5 h at room temperature while gently shaking. The membranes were then washed three times for 5–8 min each. Protein bands were detected using an ECL detection kit (Proteintech, Chicago, USA).

### Cell viability, TG, and LDH assays

Cell viability was measured using the CCK-8 assay (Beyotime Biotechnology). Intracellular TG levels were quantified with a triglyceride assay kit (Nanjing Jiancheng Bioengineering Institute, Nanjing, China), and lactate dehydrogenase assay kit in the culture supernatant was determined following the manufacturer’s protocol.

### Transcriptomic sequencing

Calf primary hepatocytes treated with 1.2 mM NEFA (N) and 1.2 mM NEFA + 75 µM choline (NC) were selected for transcriptomic sequencing. Total RNA was extracted and its concentration and purity were assessed using a Nanodrop2000 spectrophotometer (Thermo-Fisher, USA). RNA integrity was evaluated by agarose gel electrophoresis, and RNA quality was further assessed using an Agilent 2100 Bioanalyzer. The enriched mRNA was fragmented, reverse-transcribed into cDNA, and used for Library construction. PCR amplification was performed, and PCR products were purified with AM Pure XP beads. The final Library was quantified using a Qubit 2.0 Fluorometer, diluted to 1.5 ng/µL, and analyzed on an Agilent 2100 Bioanalyzer to verify insert size. The effective concentration of the library was confirmed by qRT-PCR (effective concentration > 2 nM) to ensure Library quality. After passing quality control, the Library was sequenced on the Illumina NovaSeq 6000 platform, and data processing was performed by Novogene (Beijing, China). Raw sequencing data were filtered, and the quality of the clean data was assessed by calculating Q20, Q30, and GC content.

Differential gene expression was analyzed using DESeq2 software based on the negative binomial distribution, and *P*-values were adjusted using the Benjamini–Hochberg method to control the FDR. Genes with a *P*-value < 0.05 and |log2 fold change|> 1 were considered significantly different. Genes with log2 fold change > 0 were classified as upregulated, while those with log2 fold change < 0 were classified as downregulated.

GO and KEGG pathway enrichment analyses were performed using ClusterProfiler (v3.4.4), with *P*-adjust value < 0.05 as the significant threshold.

### Statistical analyses

All experiments were performed in triplicate. Data analysis was conducted using GraphPad Prism 5 (GraphPad Software) and SPSS 26.0 (IBM SPSS Statistics, Chicago, IL, USA). Statistical analysis of cell viability, TG levels, LDH activity, and gene expression was performed using one-way ANOVA, Student's t-test, and the Duncan post-hoc test for multiple comparisons. DEGs were analyzed using a volcano plot to assess their changes across experimental groups, and gene expression patterns were visualized using a cluster heatmap. Data are presented as means ± standard error. Statistical significance was defined as *P* < 0.05, and *P* < 0.01 was considered highly significant.

## Supplementary Information

Supplementary Material 1.

## Data Availability

All data and materials are available in the paper and online supplemental files.

## Abbreviations

ABCD1: ATP binding cassette subfamily D member 1
ACC: Acetyl-CoA carboxylase
AMPK: AMP-activated protein kinase
ApoB100: Apolipoprotein B100
BP: Biological process
BSA: Bovine serum albumin
BSEP: Bile salt export pump
CC: Cellular component
ChREBP: Carbohydrate response element binding protein
CREBRF: CREB3 regulatory factor
CPT1: Carnitine palmitoyltransferase 1
CYP7A1: Cytochrome P450 family 7 subfamily A member 1
CYP27A1: Cytochrome P450 family 27 subfamily A member 1
DEG: Differentially expressed gene
FAS: Fatty acid synthase
FBS: Fetal bovine serum
FDR: False discovery rate
FOXO1: Fork head box 01
FXR: Farnesoid X receptor
GDF10: Growth differentiation factor 10
GNMT: Glycine -N- methyltransferase
GO: Gene ontology
HDL: High density lipoprotein
HNF4: Hepatocyte nuclear factor 4
HMGCR: 3-Hydroxy-3-methylglutaryl-CoA reductase
HSD17B8: Hydroxysteroid 17-beta dehydrogenase 8
IGFBP1: Insulin like growth factor binding protein 1
KEGG: Kyoto encyclopedia of genes and genomes
LDH: Lactate dehydrogenase
MAPK: Mitogen activated protein kinase
MAT: Methionine adenosyltransferase
MC: Molecular function
MTTP: Microsomal triglyceride transfer protein
NEB: Negative energy balance
NEFA: Non-esterified fatty acids
NTCP: Na^+^-dependent taurocholate transporter
PIWIL2: Piwi like RNA-mediated silencing 2
PPARα: Peroxisome proliferator-activated receptor alpha
RPC: Rumen-protected choline
RPMI: Roswell park memorial institute
SAH: S-adenosine homocysteine
SAM: S-adenosylmethionine
SDH: Succinate dehydrogenase
SPSS: Statistical package for the social sciences
SR-B1: Scavenger receptor class B type I
SREBP1: Sterol -regulatory element binding protein 1
TG: Triglycerides
TGFB3: Transforming growth factor beta 3
TGR5: G protein-coupled receptor 5
VLDL: Very low-density lipoprotein
