# Supplementary material for: Choline attenuates NEFA-induced hepatic steatosis via GNMT regulation in hepatocytes
Source: Stress Biol. 2025 Nov 14;5(1):70. doi: 10.1007/s44154-025-00264-3 (PMC12615869; doi:10.1007/s44154-025-00264-3)
Supplement: Supplementary file 1 — Supplementary Material 1. [file 44154_2025_264_MOESM1_ESM.docx]

# Supplement Table

## **Table S1.** **Sequences of primers.**

| Sepcies | Target gene | Forward (5’-3’) | Reverse (5’-3’) |
| --- | --- | --- | --- |
| Bovine | β-actin | GCTAACAGTCCGCCTAGAAGCA | GTCATCACCATCGGCAATGAG |
|  | ACCα | ATGAAGGCTGTGGTGATGGA | TGGTGGTCTTGCTGAGTTGA |
|  | CPT1 | GGAATCTGTGAAGCCTCTTATGAA | GCCTGGATGTGAGTCGGTAT |
|  | FAS | ACAGCCTCTTCCTGTTTGACG | CTCTGCACGATCAGCTCGAC |
|  | GNMT | CAGCTCTACATCGGGGACACC | CACTGGCATCCACGCTTGTC |
|  | Myc | ATACGGAACTCTTGCGCCTA | CCAAGGTTGTGAGGTTGTTCA |
|  | PGC-1α | GACAGCTCCACCAACATCCA | TCCCTGCTTGCACATCTCTC |
| Human | β-actin | GCACTCTTCCAGCCTTCCTT | AATGCCAGGGTACATGGTGG |
|  | ACCα | GGATCCGGCGCCTTACTT | CTCCGATCCACCTCATAGTTGAC |
|  | ApoB100 | GGAATGGAACCACCTCAGCA | TTCACTCCCATGCTCCGTTC |
|  | BSEP | CAGACACTGGCGTTTGTTGG | TTTGAGCGGAGGAACTGGAC |
|  | CPT1-α | GTCCCGGCTGTCAAAGACA | CCGACAGCAAAATCTTGAGCA |
|  | CYP7A1 | GAGAAGGCAAACGGGTGAAC | GAGAAGGCAAACGGGTGAAC |
|  | CYP27A1 | CCTTCGTCAGATCCATCGGG | GGGCCTCCATATCTTCGAGC |
|  | FAS | CCGAGGAACTCCCCTCAT | GCCAGCGTCTTCCACACT |
|  | GNMT | GCGTGTGGCAGCTGTATATC | CGTCACACTGAAGCCCTCTT |
|  | HMGCR | TAGATTCGTTTCCCCAGG | TAGATTCGTTTCCCCAGG |
|  | MTTP | TCTCTACTCGGGTTCTGGCATTCTA | GCTGCGATTAAGGCTTCCAGTC |
|  | NTCP | TGCACCATAGGGATCGTCCT | GGCAGAGAGAACTGTGACGG |
|  | PGC-1α | CTTTGGAGGCAAGCAAGCAG | GCTTGACTGGGATGACCGAA |
|  | PPARα | TTTGCCATGAAGTTCAATGCA | AGCAGCCACAAAAAGGGAGAT |
|  | SRB1 | GTCTACCCACCCAACGAAGG | TTCTGACAACACAGGGTCGG |

## **Table S2.The reaction system and condition of RT-PCR.**

| **Reagent name** | **Volume (μL)** |
| --- | --- |
| 2× SYBR® Green Pro Taq HS Premix | 5.0 |
| Forward primer (10μM) | 0.5 |
| Reverse primer (10μM) | 0.5 |
| cDNA | 4.0 |
| Reaction conditions: 95℃ 30 s; 2 95℃ 5 s, 60℃ 30s (40 cycles)( two step algorithm)  Dissociation stage: 95℃ 10 s, 65℃ 60 s, 97℃ 1s | |

## **Table S3. Statistics for quality control of sequencing data.**

| Sample name | Raw reads | Clean reads | Error rate（%） | Q20（%） | Q30（%） | GC content（%） |
| --- | --- | --- | --- | --- | --- | --- |
| N1 | 46323330 | 45360936 | 0.03 | 97.84 | 93.98 | 52.23 |
| N2 | 46715910 | 45617864 | 0.03 | 97.91 | 94.19 | 52.22 |
| N3 | 47871972 | 46808016 | 0.03 | 97.76 | 93.76 | 52.28 |
| N4 | 46134472 | 45069650 | 0.02 | 98.03 | 94.43 | 52.41 |
| N5 | 48054418 | 47070780 | 0.03 | 97.75 | 93.83 | 52.88 |
| NC1 | 44485216 | 43447166 | 0.02 | 98.01 | 94.40 | 52.76 |
| NC2 | 45718956 | 44800448 | 0.02 | 97.98 | 94.36 | 52.68 |
| NC3 | 46660124 | 45597338 | 0.03 | 97.84 | 94.05 | 52.62 |
| NC4 | 48184958 | 47133694 | 0.03 | 97.93 | 94.20 | 52.76 |
| NC5 | 43987120 | 42939980 | 0.02 | 98.01 | 94.41 | 52.60 |

Abbreviations: N = 1.2 mM NEFA group; NC = 1.2 mM NEFA + 75 μM choline group.

## **Table S4. Difference gene statistics.**

| Group | All DEGs | Up | Down | Threshold |
| --- | --- | --- | --- | --- |
| N vs NC | 480 | 249 | 231 | *P*-value <= 0.05，\|log2（Fold Change）\|> 0 |

Abbreviations: N = 1.2 mM NEFA group; NC = 1.2 mM NEFA + 75 μM choline group.

# **
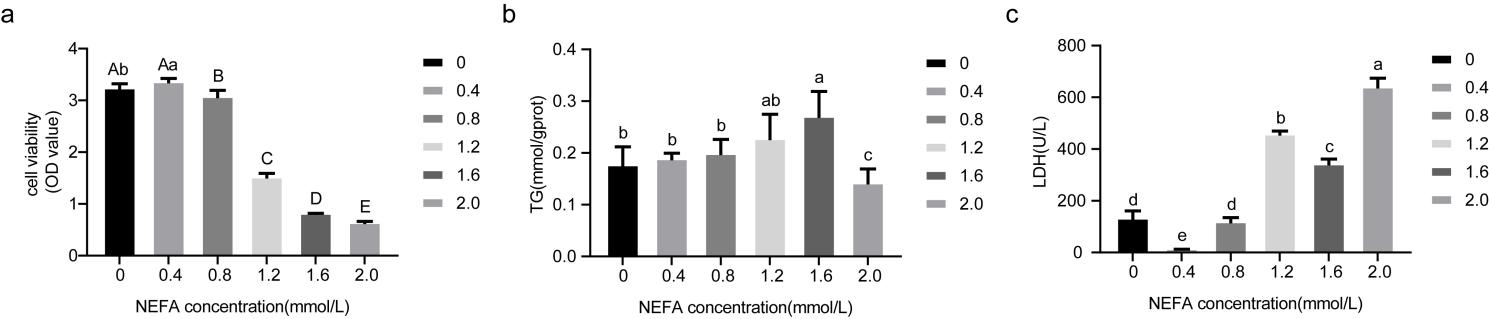
**Supplement Figure

## **Figure S1.** **Effects of different concentrations of NEFA on cell viability, LDH activity, and TG content in LO2 liver cells.** (a) Cell viability (CCK-8); (b) LDH activity; (c) TG content. Each treatment was performed in triplicate with independent biological replicates. Data are presented as mean ± SEM. Different lowercase letters indicate *P* < 0.05, different capital letters indicate *P* < 0.01 (Duncan’ test).


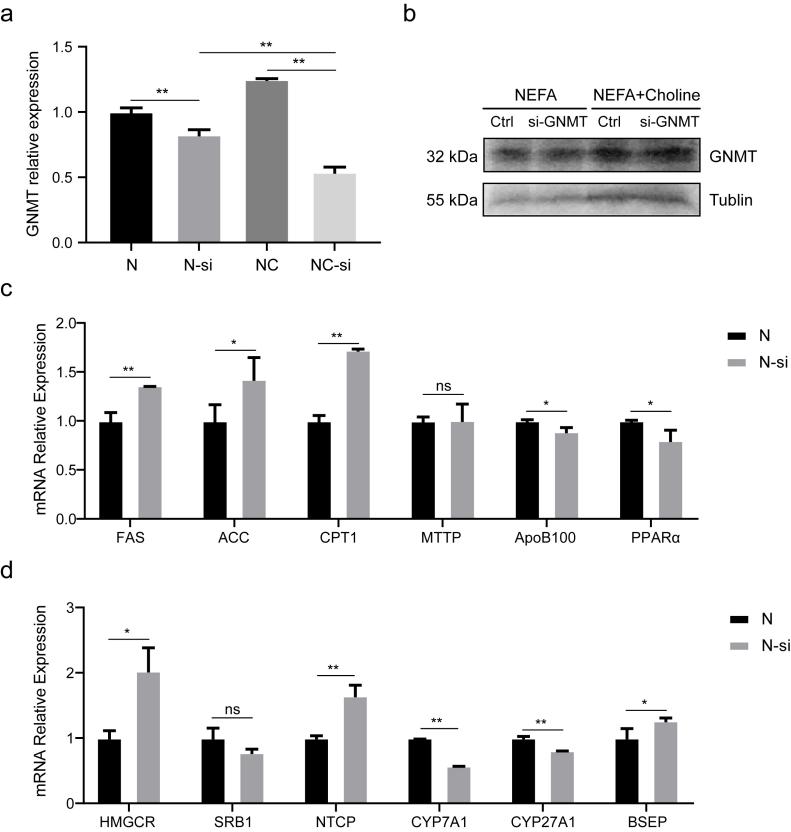
**Figure S2. Effects of GNMT knockdown on lipid and bile acid metabolism in LO2 hepatocytes under NEFA treatment.**

(a-b) Validation of GNMT knockdown efficiency at the mRNA and protein levels in LO2 cells treated with 1.6 mM NEFA. (c) Relative mRNA expression of genes involved in lipid synthesis, oxidation, and transport (FAS, ACC, CPT1, MTTP, ApoB100, and PPARα). (d) Relative mRNA expression of genes involved in cholesterol and bile acid metabolism (HMGCR , SRB1 , NTCP , CYP7A1, CYP27A1, and BSEP). Each treatment was performed in triplicate with independent biological replicates. Data are presented as mean ± SEM.

Abbreviations: N = 1.6 mM NEFA; NC = 1.6 mM NEFA + 50 μM choline; N-si = 1.6 mM NEFA + GNMT-siRNA; NC-si = 1.6 mM NEFA + 50 μM choline + GNMT-siRNA.

Statistical significance: *P < 0.05, **P < 0.01, ns = not significant (*P* > 0.05) (Student's t-test, and Duncan’ test).

# Supplement Material 1. Configuration of cholinine-free RPMI1640 medium

First, the instruments used in the preparation process were sterilized and dried, 2.0g sodium bicarbonate (NaHCO_3_) solid and 10.4 g of powder of the customized culture-medium (purchased from Gibco) free of chyline, and 1 L of sterilized ultra-pure water was added to the beaker for stirring and dissolution (about 2-3 h), and covered with plastic wrap to prevent evaporation. After no particle residue was observed, 3.6% of bovine serum albumin (BSA) (Amersco) was added and continued to be stirred until completely dissolved. The pH value was adjusted, and the mixture was filtered and subpacked with a 0.22 micron filter in a super-clean table and stored at 4℃.

# Supplement Material 2. Isolation of calf primary hepatocytes

Firstly, the liver caudate process of A day-old healthy calf was collected from a dairy farm and quickly put into a sterile beater prepared in advance. A 50mL sterile syringe was inserted into the portal vein for perfusion of perfusion fluid A pre-cooled at 4 ° C in advance. The perfusion rate was maintained at 50mL/min. The whole liver tissue was immersed in A sterile container of perfusion solution A (140 mM NaCl, 6.7 mM KCl, 2.5 mM Glucose, 10 mM HEPES, 0.5 mM EDTA, pH7.4, sterile filtration), placed in a freezer, and quickly transferred to the laboratory. The liver tissue was placed in a sterile tray on the aseptic operation table, and perfusion solution B (140 mM NaCl, 6.7 mMKCl, 2.5 mM Glucose, 30 mM HEPES, 5 mM CaCl2, pH 7.4, aseptic filtration) pre-warmed at 37℃ was used for the second step of perfusion, and the effluent liquid became clear for about 3min. Then, the perfusion solution C containing type IV collagenase pre-heated at 37℃ was used for the third step of digestive perfusion, and the perfusion rate was maintained at 50mL/min to ensure adequate digestion. When the effluent became turbid, the perfusion was stopped immediately, and the liver tissue was quickly cut up in a petri dish. Then, digestion was terminated with serum, filtered with cell sieve (80 mesh and 200 mesh), poured into a centrifuge tube, added serum-free 1640 base medium, centrifuged at 4℃50g for 2min, and centrifuged twice. Finally, the adherent medium was added for re-suspension, inserted into a 6-well plate, the cell density was adjusted to 1.5×106 cells /mL, and placed into a constant temperature incubator containing 5%CO2 at 37℃ for 4h, after adherent culture, the liquid was changed, and the growth medium was cultured, and the medium was changed every 24h.
